# P05 Evaluation of antibiotic resistance in *Gardnerella vaginalis* isolates

**DOI:** 10.1093/jacamr/dlag102.011

**Published:** 2026-06-26

**Authors:** S Parikh, M Vidwans, B Haripara, S Venkataraman

**Affiliations:** Harley Street Pathology Services, London, UK; Harley Street Pathology Services, London, UK; Harley Street Pathology Services, London, UK; Harley Street Pathology Services, London, UK

## Abstract

**Background:**

*Gardnerella vaginalis* is a facultatively anaerobic Gram-variable rod that is involved, together with many other bacteria, mostly anaerobic, in bacterial vaginosis (BV) in some women as a result of a disruption in the normal vaginal microflora.

**Objectives:**

To check antibiotic sensitivity with various commonly used antibiotics active against anaerobic bacteria.

**Methods:**

From July 2024 to October 2024, we assessed 50 patients with BV who underwent gynaecological examinations at Harley Street Fertility Clinic. *G. vaginalis* isolates were obtained from High vaginal swabs and semen samples and identified using their characteristic colony morphology and biochemical tests- RapID^™^ NH System (Thermo Scientific). Subsequently, clinical isolates were evaluated for antimicrobial susceptibilities *in vitro* to metronidazole, co-amoxiclav (amoxicillin with clavulanic acid), penicillin, ciprofloxacin, doxycycline, meropenem, ertapenem, azithromycin and piperacillin/tazobactam using the disc diffusion methodology on *Gardnerella* agar. McFarland suspension 2.0 was prepared with 0.85% saline and incubated in an anaerobic jar with an anaerobic sachet for 24 h.

**Results:**

In our study, we successfully isolated *G. vaginalis* in all specimens. Most of the samples showed moderate to heavy growth of the organism. Mostly, *G. vaginalis* was the only organism isolated. *G. vaginalis* demonstrated complete resistance to metronidazole at both 5 and 50 μg concentrations in all patient isolates, apart from the ATCC reference strain 14018, which remained susceptible. Co-amoxiclav showed consistent sensitivity against *G. vaginalis*, with inhibition zone diameters ranging from 27 to 40 mm. Susceptibility was also observed for penicillin (20–32 mm), doxycycline (25–37 mm), azithromycin (24–37 mm), piperacillin/tazobactam (Tazocin) (24–40 mm), meropenem (20–35 mm), and ertapenem (24–40 mm). All patient isolates exhibited resistance to Metronidazole and variable resistance to ciprofloxacin. Despite metronidazole being widely used for the treatment of anaerobic infections, the findings of this study indicate that co-amoxiclav is the most effective antimicrobial agent against *G. vaginalis*. Other effective antibiotics include doxycycline, azithromycin, piperacillin/tazobactam, and carbapenems (meropenem and ertapenem).

**Conclusions:**

Antibiotic resistance seems to be spreading ever wider in the world and poses a well-established risk to public health. Our study demonstrates widespread (almost universal) resistance of *G. vaginalis* to metronidazole, which is the first-line therapy for BV. Clinicians need to be made aware of this state of affairs, and guidelines modified to treat BV effectively.

